# The application of omics technologies in the functional evaluation of inulin and inulin-containing prebiotics dietary supplementation

**DOI:** 10.1038/nutd.2015.35

**Published:** 2015-11-30

**Authors:** M Tsurumaki, M Kotake, M Iwasaki, M Saito, K Tanaka, W Aw, S Fukuda, M Tomita

**Affiliations:** 1 Faculty of Environment and Information Studies, Keio University, Fujisawa, Japan; 2 Institute for Advanced Biosciences, Keio University, Tsuruoka, Japan; 3 Graduate School of Media and Governance, Keio University, Fujisawa, Japan

## Abstract

Inulin, a natural renewable polysaccharide resource produced by various plants in nature, has been reported to possess a significant number of diverse pharmaceutical and food applications. Recently, there has been rapid progress in high-throughput technologies and platforms to assay global mRNA, proteins, metabolites and gut microbiota. In this review, we will describe the current status of utilizing omics technologies of elucidating the impact of inulin and inulin-containing prebiotics at the transcriptome, proteome, metabolome and gut microbiome levels. Although many studies in this review have addressed the impact of inulin comprehensively, these omics technologies only enable us to understand physiological information at each different stage of mRNA, protein, metabolite and gut microbe. We believe that a synergistic approach is vital in order to fully illustrate the intricate beauty behind the relatively modest influence of food factors like inulin on host health.

## Introduction: what is inulin?

**Figure 1:**
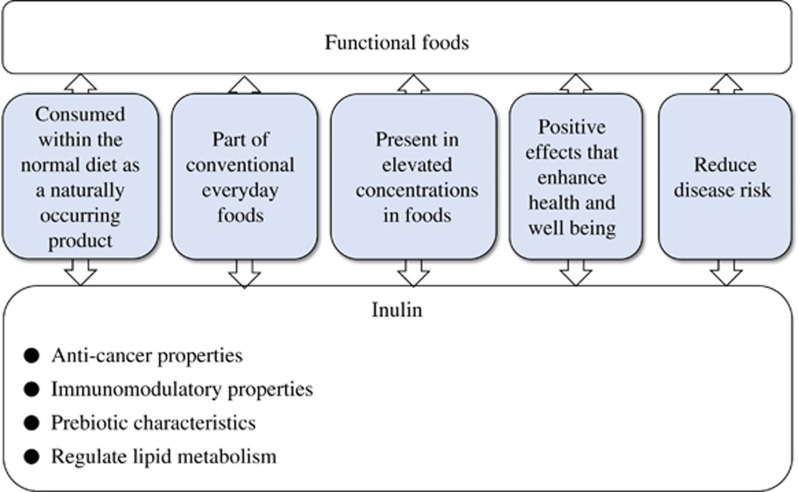
Inulin or inulin-containing prebiotic fiber as a functional food. Inulin is a natural renewable polysaccharide resource produced by various plants in nature. Plant bulbs of *Asteraceae*: *Jerusalem artichoke* and chicory (*Cichorium intybus*) use it as a means for storing nutrients. Inulin is also included in dandelion and dahlia. Inulin fulfills the requirements of functional foods in the following manner: being a part of conventional everyday foods, to be consumed with the normal/usual diet as naturally occurring (as opposed to synthetic) components, sometimes in increased concentrations or present in foods that would not normally supply them, and having positive effects on target functions that may enhance health and well-being, as well as reduce disease risk.

α-D-glucopyranosyl-[α-D-fructofuranosyl](n-1)-D-fructofuranoside or β-glucopyranosyl-[D-fructofuranosyl](n-1)-D-fructofuranoside, also more commonly known as inulin, is a natural renewable polysaccharide resource produced by various plants in nature with a significant number of diverse pharmaceutical and food applications. Plant bulbs of *Asteraceae* use it as a means for storing nutrients.^1^ Owing to its excellent nutritional properties, its use in food products has increased in recent years. Sometimes it is used in place of sugar, fat and flour. Inulin is included in *Jerusalem artichoke*, chicory (*Cichorium intybus*), dandelion and dahlia.^2^ Inulin is sometimes used as a fat or sugar replacement in the food industry;^3, 4, 5, 6, 7^ however, it also has important pharmaceutical applications, as an excipient or a stabilizer, and as an injectable for the clinical measurement of kidney function.^6, 8^ In addition, inulin possesses interesting biological effects, being a potent complement pathway activator when in a particulate form and having anticancer^9, 10^ and immunomodulatory properties^11, 12, 13, 14^ (Figure 1).

## Inulin as a prebiotic and functional food

Inulin is resistant to hydrolysis by small human gut digestive enzymes, as the anomeric C2 in fructose monomers is beta-configured and forms β 2–1 glyosidic linkages. As such, inulin is categorized as a ‘non-digestible' oligosaccharide.^15^ As reported by a large number of *in vitro* and *in vivo* studies, inulin is fermented by bacteria colonizing the large bowel with lactate and short-chain fatty acids (SCFAs), mainly acetate, as end products of the fermentation process.^16^ Furthermore, well-designed human studies have shown that inulin dietary intervention induced significant changes in the composition of human fecal microbiota, concluding that inulin possesses prebiotic properties.^17, 18, 19^ Apart from being a prebiotic, inulin also belongs to the category of functional foods, which, by reference to the European consensus,^20^ comprises several unique characteristics such as: being a part of conventional everyday foods, to be consumed with the normal/usual diet as naturally occurring (as opposed to synthetic) components, sometimes in increased concentrations or present in foods that would not normally supply them, and having positive effects on target functions that may enhance health and well-being, as well as reduce disease risk. As stipulated by the Japanese Ministry of Health, Labour and Welfare, oligofructose, an inulin-containing, prebiotic food product, is classified as a functional food or, as specified by ‘Food for Specified Health Uses', as a food that can modify gastrointestinal conditions (http://www.mhlw.go.jp/english/topics/foodsafety/fhc/02.html). Inulin is indeed present in commonly consumed plants and can be added to normal food products. In addition, inulin dietary intervention can regulate the key physiological functions such as lipid metabolism;^21^ they modulate the composition of gut microbiota, which has a major role in gastrointestinal physiology, and, finally, might have a role in reducing the risk of colon cancer.^22^

## Nutrigenomics in food and nutrition research

**Figure 2:**
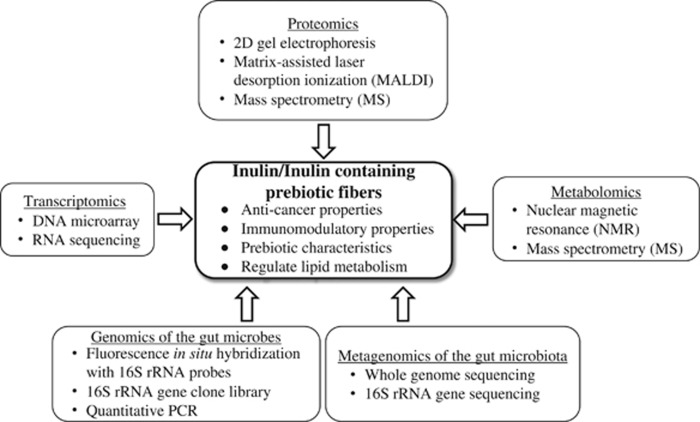
Application of omics technologies in functional evaluation of inulin and inulin-containing prebiotic fibers. Inulin dietary intervention can regulate the key physiological functions such as lipid metabolism and composition of gut microbiota, and can reduce cancer risk. Omics technologies such as transcriptomics, proteomics, metabolomics, metagenomics of the gut microbiota and genomics of the gut microbes have been employed to elucidate the functionality of inulin supplementation.

In recent years, there has been rapid progress in high-throughput technologies and platforms to assay global mRNA, proteins, metabolites and gut microbiota. As a consequence, today's researchers of food and nutrition science have been witnessing a rapid expansion of nutrigenomics, also known as nutriomics, comprising transcriptomics, proteomics, metabolomics and metagenomics.^23^ Nutrigenomics is a discipline in which all available lines of information about the genome and other biological molecules are effectively utilized to unveil every detail of the interactions between diets and the human body.^24, 25^ In this review, we describe the current status of omics approaches toward elucidating the molecular mechanisms of inulin supplementation *in vivo* (Figure 2 and Table 1).

## Transcriptomic evaluation of inulin supplementation

Transcriptomics is the most widely employed omics technology, as compared with others in food research, because of the many merits of the DNA microarray technology, which include the comprehensiveness of the gene expression data, established protocols and high reliability and reproducibility of the data.^26, 27, 28, 29^ Another upcoming popular approach in transcriptomics is RNA-sequencing, also known as whole-transcriptome sequencing.^30^ As compared with microarrays, RNA-sequencing at sufficient coverage captures a wider range of expression values. As a digital measure (count data), it scales linearly even at extreme values, whereas microarrays only show saturation of analog-type fluorescent signals.^31^ RNA-sequencing further provides information on RNA splice events, which are not readily detected with standard microarrays.^32^

Recently, Sevane *et al.*^33^ reported about the differences in the hepatic transcriptome profiles between chickens supplemented with inulin (5 g of inulin per kg diet) and controls for 34 days. Overall, 148 differentially expressed genes were identified because of inulin dietary supplementation, and Kyoto Encyclopedia of Genes and Genomes pathway visual analysis identified that three immune-system-related genes, *tumor necrosis factor receptor superfamily member 1B*, *acyl-CoA synthetase long-chain family member 6* and *peroxisome proliferator-activated receptor, alpha*, were upregulated with inulin supplementation. Comprehensive gene expression analyses have revealed that inulin supplementation, while reinforcing the immune status of animals, and fostering the production of long-chain fatty acids, is also involved in chicken growth and performance. This study is useful in the application of promoting the use of prebiotics on chicken diets as a useful alternative to antibiotics for producing chickens with a healthier meat lipid profile and also for improving performance and general immunity in poultry farming. In addition, Parnell and Reimer^34^ identified possible mechanisms through which prebiotic fibers improve serum lipids. Lean and obese male JCR:La-cp rats were fed with the following three diets: 0, 10 or 20% prebiotic fiber comprising 1:1 mix of inulin and oligofructose for 10 weeks. Both diets of prebiotic fiber significantly lowered serum-cholesterol levels by 24% in the obese hyperlipidemic rats. Expression levels of hepatic genes primarily associated with cholesterol metabolism, *cholesterol 7-alpha-monooxygenase*, *hydroxymethylglutaryl-CoA reductase*, *lecithin-cholesterol acyltransferase* and *sterol regulatory element-binding protein 2*, were significantly decreased in rats fed on prebiotic fibers. There was also an increase in cecal digesta as well as an upregulation of genes involved in cholesterol synthesis and bile production. As such, prebiotic fibers containing inulin may be considered as a potential dietary intervention for hypercholesterolemia.^34^ In another study conducted by the same group, dose-dependent effects of prebiotics (inulin and oligofructose) on gut satiety hormones, energy expenditure, gastric-emptying and gut microbiota were investigated. Male lean and obese JCR:LA-cp rats were divided into groups and administered the following diets: lean 0% fiber (LC), lean 10% fiber (LF), lean 20% fiber (LHF), obese 0% fiber (OC), obese 10% fiber (OF) or obese 20% fiber (OHF) for 10 weeks. Biochemical parameters measured include body composition, gastric-emptying, energy expenditure, plasma satiety hormone concentrations and presence of gut microbiota detected using quantitative PCR. It was observed that cecal proglucagon and peptide YY mRNA levels throughout the entire gastrointestinal tract were upregulated twofold in the LF, OF and OHF groups and threefold in the LHF group. Ghrelin *O*-acyltransferase mRNA levels of the fundus in the stomach were higher in obese rats as compared with lean rats, and had lowered gene expression in the OHF group. It was revealed that prebiotics regulated several of the mechanisms including food intake, satiety hormones and alterations in gut microbiota in a dose-dependent manner. As such, the results of this study conclude that the combined effects of prebiotics may have a therapeutic potential for obesity.^35^ Jerusalem artichoke (JA), which mainly comprises inulin, has been reported to potentially attenuate lipid disturbances and insulin resistance; however, the underlying mechanisms are not well understood. In a study by Chang *et al.*,^36^ the physiological responses and mechanisms of JA intervention were elucidated via a comprehensive transcriptome analysis. Wistar rats were fed a control diet (CT), a 60% fructose-enriched diet (FRU) or a FRU with 10% JA (*n*=6–7) for 4 weeks. Dietary JA supplementation significantly improved insulin resistance and hepatic triglyceride accumulation. Transcriptomic profiling of the liver revealed that FRU significantly altered the expression of *malic enzyme 1, associated with fatty-acid synthesis; decorin, related to fibrosis; cytochrome P450, family 1, subfamily a, polypeptide 2; and nicotinamide phosphoribosyl transferase* associated with inflammation, whereas dietary JA supplementation significantly modulated the expression of these genes. As such, it was proposed that 10% JA supplementation may be beneficial for the prevention of the onset of type 2 diabetes and non-alcoholic fatty liver disease. Wu and Chen^37^ attempted to compare and investigate the effects of konjac glucomannan (KGM). Male Sprague–Dawley rats were fed a high-fat (25% corn oil, w/w), fiber-free diet or that supplemented with either KGM or inulin fiber (5%, w/w) for 4 weeks, after which the index of pro-oxidative status, malondialdehyde levels, blood lymphocyte DNA damage, colonic mucosa, hepatic concentrations of antioxidant enzymes (glutathione peroxidase, superoxide dismutase and catalase) and the plasma antioxidant levels were measured. It was observed that the incorporation of KGM and inulin into the high-fat, fiber-free diet beneficially reduced the malondialdehyde levels of the colon and liver and DNA damage in blood lymphocytes. In addition, the gene expressions of colonic mucosa glutathione peroxidase and catalase and of hepatic superoxide dismutase and catalase were upregulated by dietary intervention of both fibers. These results suggest that *in vivo* utilization of KGM and inulin activates both the local and systemic antioxidative defense systems in rats. In a novel study by Everard *et al.*,^38^ the targets involved in host response during obesity in oligofructose-treated mice were investigated by feeding 10-week-old C57BL/6J mice CT, a CT supplemented with oligofructose (0.3 g per mouse per day) added in tap water (CT-Pre), a high-fat diet (HFD) (60% fat and 20% carbohydrates (kcal per 100 g) or a HFD supplemented with oligofructose (0.3 g per mouse per day) added in tap water (HFD-Pre) for 8 weeks. It was observed that, whereas HFD feeding significantly decreased the expression of regenerating islet-derived 3-gamma (*Reg3g*) and phospholipase A2 group-II (*PLA2g2*) in the jejunum, prebiotic dietary intervention upregulated *Reg3g* expression and improved gut homeostasis as suggested by the increase in the protein expression levels of intectin that has a main role in gut epithelial cell turnover. In addition, prebiotic supplementation also counteracted gut microbiota modulations induced by HFD-induced inflammation and related metabolic disorders.

## Proteomic evaluation of inulin supplementation

The separation, quantification and identification of proteins are the steps commonly involved in proteome analyses.^39^ In conventional protein detection methods, proteins in biological samples are separated using two-dimensional gel electrophoresis, in which separated proteins are visualized and quantified after silver or fluorescent staining. However, the differential imaging gel electrophoresis method is a more sophisticated strategy based on the same principle of separation in which proteins of different samples are pre-labeled with different fluorescent dyes. Gel-free separation usually relies on chromatography that includes two-dimensional chromatography.^40^ Recently, a novel, tandem mass spectrometry (MS)-based approach for the relative quantification of proteins, relying on isobaric tag for relative and absolute quantitation, has been reported. Isobaric tag for relative and absolute quantitation reagents are designed in a manner in which their mass is isobaric. As such, differentially labeled proteins do not differ in mass. In MS scans, the corresponding proteolytic peptides appear as single peaks.^41^ Despite the advances in quantitative proteomics, MS remains the most widely used method for the identification of proteins with evaporation of peptides and proteins using matrix-assisted laser desorption/ionization and electrospray ionization.^42^ The evaluation of inulin dietary interventions in disease prevention using proteomic analyses is yet to be reported. As such, in this section, we would focus on using proteomic technologies to analyze components and stability of the inulin compound.

Two-dimensional electrophoretic analysis of proteins from chicory root was performed before the first freezing period. After protein digestion with trypsin, the peptides were analyzed using MS (matrix-assisted laser desorption/ionization-time-of-flight/time-of-flight). From the 881 protein spots analyzed, 714 proteins corresponded to a database accession, 619 of which were classified into the following functional categories: metabolism, energy, protein synthesis, cell structure, folding and stability, proteolysis and stress response. The importance of abiotic stress response was attested, as 7 of the 21 most intense protein spots observed are known to be involved in cold acclimation, suggesting the major effect of the low-temperature period that preceded root harvesting.^43^

## Metabolomic evaluation of inulin supplementation

Metabolomics is widely used in the identification of disease biomarkers. By using nuclear magnetic resonance and/or MS, we can capture the metabolome using a wide range of analytical methods by providing robust and sensitive identification of metabolites produced by microbiota and host cells in fecal, blood, urine and tissue samples.^44^ Ranging from targeted to untargeted methods, methodologies have been reported for screening biochemical pathways, for example, central carbon metabolism, glycolysis, tricarboxylic acid cycle, amino-acid pathways, lipid pathways and selected secondary metabolism pathways.^45, 46^ These tools allow researchers to determine the effects that treatments have on the host's metabolic profile by analyzing the presence and quantity of thousands of metabolites simultaneously. There are various challenges in the metabolite analysis because of the diversity of the chemical properties of metabolites, which is larger in number than those of transcripts and proteins. In addition, another difficulty derives from the width of the abundance of metabolites. As such, metabolite analysis usually requires the use of techniques requiring a high skill level. Despite these difficulties, metabolome analysis is a powerful tool in the field of food and nutrition science.^47^

Duan *et al.*^48^ comprehensively analyzed the effects of HFD and inulin intake on the metabolite compositions of myocardium and testicle using nuclear magnetic resonance spectroscopy. Multiple univariate data analysis is a high-throughput method utilized to visualize and efficiently extract information on metabolites significantly affected by an intervention. Using multiple univariate data analysis, it was reported that HFD induced metabolic changes in rat testicles and myocardium that are involved in fatty-acid β-oxidation, together with the metabolisms of choline, amino acids, purines and pyrimidines, even before HFD resulted in significant body-weight increases. Inulin intake attenuated some of the HFD-induced metabolic changes in both myocardium (3-HB, lactate and guanosine) and testicle tissues (3-HB, inosine and betaine). These observations suggest that inulin intervened HFD-induced metabolic changes and illustrated that multiple univariate data analysis is a power-alternative method in metabolomics analysis.

De Preter *et al.*^49^ demonstrated that inulin supplementation modulates the fecal metabolite profile *in vitro*. Fecal samples obtained from healthy subjects were anaerobically incubated at 37 °C with or without increasing doses of inulin, and changes in the metabolite pattern and pH were assessed. According to their chemical classes, a total of 107 different volatile organic compounds were identified and classified. The concentrations of esters, acids and some aldehydes were significantly increased with increasing doses of inulin. On the contrary, inulin dose-dependently inhibited the formation of S-compounds. In addition, the generation of other protein fermentation metabolites such as phenolic compounds was inhibited in the presence of inulin.

## Gut microbiome evaluation of inulin supplementation via metagenomic approach

The gut microbiota consists of all the microorganisms inhabiting the gastrointestinal tract. In most mammals, the gut microbiota is dominated by four bacterial phyla, *Firmicutes*, *Bacteroidetes*, *Actinobacteria* and *Proteobacteria*, which perform roles that define the health of the host.^50^ The gut is mainly populated by bacteria amounting to ~100 trillion cells, which is approximately threefold larger than the number of human body cells^51^ and has extensive metabolic capabilities.^50^ Thus, the gut microbiota that creates the unique gut ecosystem together with the host eukaryotic cells consists of prokaryotic cells and is frequently referred to as a measurable and functional organ.^52^ In recent years, the gut microbiome can be comprehensively analyzed using next-generation whole-DNA sequencing and 16S rRNA gene sequencing.^53, 54, 55^ One of the advantages is that microbial community profiling using 16S rRNA genes allows us to gain deep views into hundreds of microbial communities simultaneously.^56^ According to the lifestyle and nutritional status of the host, the bacterial communities vary in composition along the digestive tract and adapt through life.^57^

In a novel study by Everard *et al.*, to investigate the targets involved in host response during obesity in oligofructose-treated mice, 10-week-old C57BL/6J mice were fed a CT, a CT supplemented with oligofructose (0.3 g per mouse per day) added in tap water (CT-Pre), a HFD (60% fat and 20% carbohydrates (kcal per 100 g) or a HFD supplemented with oligofructose (0.3 g per mouse per day) added in tap water (HFD-Pre) for 8 weeks. Results from deep metagenomic sequencing revealed that HFD and prebiotic treatment significantly altered the gut microbiome at different taxonomic levels. Distinct profiles for the HFD, Pre, HFD-Pre and CT groups were observed from the functional analyses based on the occurrence of clusters of orthologous groups of proteins. Finally, the modulations in gut microbiota induced by prebiotic intervention counteracted HFD-induced inflammation and related metabolic disorders.^38^

## Genomic evaluation of the impact of inulin supplementation on the gut microbes

In this review, we have highlighted several studies that have analyzed the impact of inulin supplementation using various omics technologies such as transcriptomics, proteomics, metabolomics and metagenomics. Genomic technologies, for example, quantitative polymerase chain reaction,^58^ fluorescent *in situ* hybridization^59^ and *in situ* hybridization with 16S rRNA-targeting probes,^60^ are also some of the popular omics technologies that have been used in the interpretation of inulin intervention. Another genomic approach includes the application of 16S rRNA gene clone library sequencing using capillary sequencer.^61^

In a study by Ramirez-Farias *et al.*,^62^ variations in the fecal microbiota composition were analyzed using real-time PCR in human volunteers after inulin ingestion (10 g per day) for 16 days in comparison with a control period without any supplement intake. The prevalence of most bacterial groups examined was not affected. However, there was a significant increase in *Faecalibacterium prausnitzii* after inulin intake. Using clone library analysis, the composition of the genus *Bifidobacterium* was studied in four of the volunteers. There were between three and five *Bifidobacterium* spp. observed in each volunteer. *B. adolescentis* and *B. longum* were present in all the volunteers and *B. pseudocatenulatum*, *B. animalis*, *B. bifidum* and *B. dentium* were also observed. Using real-time PCR, the four most prevalent *Bifidobacterium* spp., *B. adolescentis*, *B. longum*, *B. pseudocatenulatum* and *B. bifidum*, in volunteers carrying detectable levels of bifidobacteria and *B. adolescentis* showed the strongest response to inulin consumption, significantly increasing from 0.89 to 3.9% of the total microbiota. In addition, the amount of *B. bifidum* was remarkably increased from 0.22 to 0.63% in the five volunteers for whom this species was present. The effects of inulin on the gut microbiota were investigated using fluorescent *in situ* hybridization in growing pigs. For this study, pigs (*n*=8 per group) were assigned to two types of basal diets (wheat and barley or corn and wheat gluten) with or without 3% inulin for 3 and 6 weeks to evaluate whether naturally occurring dietary fibers influence the inulin effect. Bifidobacteria were observed in less than half of the pigs and in pigs that were administered inulin-containing diets, higher colonic bifidobacteria and lower total colonic SCFA concentrations due to reduced acetate, but higher proportions of colonic butyrate were observed. Inulin did not stimulate increase in lactobacilli and bifidobacteria numbers irrespective of the basal diet, although 20–50% of inulin was degraded in the jejunum. As such, inulin supplementation affected gut luminal SCFA and the number of pigs harboring bifidobacteria.^63^ In a clinical study to assess the tolerance and effectiveness of a prebiotic inulin/partially hydrolyzed guar gum mixture (I-PHGG) for the treatment of constipation in females, as well as its influence on the composition of gut microbiota and production of short-chain fatty acids, female health volunteers were randomized to treatment with I-PHGG or placebo. Treatment consisted of 3-week supplementation with 15 g per day I-PHGG (fiber group) or maltodextrin (placebo group). Changes in fecal bacterial population and SCFAs were assessed using real-time PCR and gas chromatography, respectively. Consumption of I-PHGG produced clinical results comparable to placebo in constipated females, but had additional protective effects on gut microbiota by decreasing the amount of pathological bacteria of the *Clostridium* genera.^64^ As reported by Duncan *et al.*, duplicate anaerobic fermentor systems were used to examine changes in a community of human fecal bacteria supplied with different carbohydrate energy sources and a panel of group-specific fluorescent *in situ* hybridizationprobes targeting 16S rRNA sequences were used. It was reported that the fermentors supported growth of a lower proportion of Gram-positive anaerobes related to *Faecalibacterium prausnitzii*, *Ruminococcus flavefaciens*–*R. bromii*, *Eubacterium rectale*–*Clostridium coccoides* and *E. cylindroides* and a greater proportion of *Bacteroides* as compared with the starting fecal inoculum. Inulin from dahlia resulted in a significant increase in the number of bacteria related to *R. flavefaciens–R. bromii* and *E. cylindroides*. *Roseburia*-related strain A2-183 F was able to grow on all substrates, despite the fact that it was unable to utilize complex carbohydrates in pure culture, and it was assumed that this organism survived by cross-feeding. In contrast, *Roseburia gutis* L1-82 R and *Eubacterium sp.* strain A2-194 R survived less well, despite the fact that they were able to utilize polysaccharides in pure culture, except that A2-194 R was stimulated 100-fold by inulin. This suggests that many low-G+C-content Gram-positive anaerobes may be selected against during *in vitro* incubation, although several groups were stimulated by inulin.^65^ In another study, the prebiotic potential of inulin was compared against arabinoxylan oligosaccharides in two simulators of the human gut microbial ecosystem. Microbial breakdown of both oligosaccharides and SCFA production was colon compartment-specific, with ascending and transverse colon being the predominant site of inulin and arabinoxylan oligosaccharide degradation, respectively. Inulin dietary intervention slightly affected concentrations of lactate, propionate and butyrate. Using denaturing gradient gel electrophoresis, it was also revealed that inulin supplementation modulated microbial metabolism by modifying the microbial community composition. The results indicate that inulin has a lower potency than arabinoxylan oligosaccharide to shift part of the sugar fermentation toward the distal colon parts. Furthermore, as AXOS has a stronger propionate-stimulating effect, it may be suitable for lowering cholesterol and beneficially affecting host lipid metabolism as a prebiotic candidate.^66^ As reported by Kleessen *et al.*, one of four following treatments: (1) commercial standard diet as a control (Con); (2) Con+50-g short-chain oligofructose per kg (OF); (3) C+50-g long-chain inulin per kg (lcIN); or (4) Con+50 g OF-lcIN/kg (Mix OF-lcIN) were assigned to germ-free rats inoculated with human fecal microbiota. At the end of the treatment period, 16S rRNA-targeted probes applied in *in situ* hybridization were used to observe the changes in bacterial population groups in response to feeding these diets. It was observed that there were larger numbers of cecal, colonic and fecal bacteria of the *C. coccoides–E. rectale* cluster in rats that fed on Mix OF-lcIN- and lcIN-containing diets, whereas OF alone did not affect this bacterial group in the cecum, colon or feces. Higher amounts of lactobacilli were found in cecal and colonic contents of Mix OF-lcIN-fed rats and in feces of OF-fed rats compared with Con. Mix OF-lcIN and OF resulted in significantly smaller numbers of cecal, colonic and fecal bacteria belonging to the *C. histolyticum* and *C. lituseburense* as compared with Con. Counts of *Bacteroides–Prevotella* and *Enterobacteriaceae* were similar between the groups. OF- and/or lcIN-containing diets significantly increased the butyrate concentration and its relative molar proportion in cecal and colonic contents. The results of this study revealed that there was higher fecal concentration of butyrate in only lcIN-containing diets than in Con. In addition, higher molar proportions of fecal butyrate were observed with all diets that had been supplemented with OF and/or lcIN. As such, the incorporation of fermentable, indigestible fructans may be beneficial to gastrointestinal health by providing SCFAs, stimulating the proliferation of bifidobacteria or lactobacilli and suppressing potential pathogenic organisms in the gut.^67^

## Adverse effects of high doses of inulin

Although we have mainly reviewed studies highlighting the beneficial properties of inulin supplementation, it is reported that high dosage of inulin intake has several side effects. The effects of daily intake of 14 g inulin added to a low-fat spread on fasting blood lipids and gastrointestinal symptoms were investigated in 64 young healthy women in a randomized double-blind crossover study. Inulin showed no effect on blood lipid concentrations. Interestingly, gastrointestinal symptoms assessed with questionnaires showed that during the inulin intake period there was a significantly higher degree of gastrointestinal symptoms such as discomfort from flatulence, bloatedness, stomach and gut cramps and rumbling in the stomach and gut than in the control period.^68^ Bonnema *et al.*^69^ conducted a doubled-blind, randomized, placebo-controlled and crossover study on the gastrointestinal tolerance of two inulin fibers: shorter-chain-length oligofructose and native inulin at 5- and 10-g doses on 26 healthy men and women aged 18–60 years. It was observed that doses up to 10 g per day of native inulin and up to 5 g per day of oligofructose were well tolerated.

## Conclusions

Although many studies in this review have addressed the impact of inulin solely at the transcriptome, proteome, metabolome and gut microbiome level, they should be interpreted with reservation that these omics technologies individually enable us to understand physiological information at each different stages of mRNA, protein, metabolite and gut microbe. However, a synergistic omics approach is of significant importance as no single omics methodology can fully illustrate the intricate beauty behind the relatively modest influence of food factors such as inulin, as addressed in this review. In addition, as the gastrointestinal tract is a complex ecosystem that harbors various microorganisms that influence many aspects of health, including inflammatory bowel disease, cancer and obesity in adults.^52^ The intricate interaction among gut microbiota-derived metabolites, the gut microbiota itself and the host immune system is transmitted via a large array of signaling pathways that extend beyond the immune system. Conjointly, the direct chemical interactions between gut microbiota and the host and the immune-mediated signaling mechanisms influence various organs such as the gut, skeletal muscle, liver and the brain. These complex inter-relationships come together to form a series of host–microbe metabolic axes.^70^ As such, integrating metagenome, metabolome, proteome and transcriptome information on a systems biology-wide approach would also allow us to better understand this interplay between inulin, inulin-containing nutraceuticals and host metabolism.

## Figures and Tables

**Table 1:** Application of omics technologies in the functional evaluation of inulin and inulin-containing prebiotics dietary supplementation

*No.*	*Type of omics evaluation*	* Summary of results*	*Reference*
1	Transcriptomics	Immune-system-related genes, *tumor necrosis factor receptor superfamily member D16*, *acyl-CoA synthetase long-chain family member 6* and *peroxisome proliferator-activated receptor, alpha*, were upregulated	^(33)^
		Inulin supplementation is involved in chicken growth and performance while reinforcing the immune status of animals and fostering the production of long-chain fatty acids	
		Addition of prebiotics on chicken diets may be an useful alternative to antibiotics for improving performance and general immunity in poultry farming	
2	Transcriptomics	Prebiotic fiber diets significantly lowered serum-cholesterol levels	^(34)^
		Expression levels of hepatic genes primarily associated with cholesterol metabolism*, cholesterol 7-alpha-monooxygenase*, *hydroxymethylglutaryl-CoA reductase*, *lecithin-cholesterol acyltransferase* and *sterol regulatory element-binding protein 2*, were significantly decreased in rats fed on prebiotic fibers	
		Increase in cecal digesta as well as an upregulation of genes involved in cholesterol synthesis and bile production	
		Prebiotic fibers containing inulin may be considered as a potential dietary intervention for hypercholesterolemia	
3	Transcriptomics	Cecal proglucagon and peptide YY mRNA levels throughout the entire gastrointestinal tract were upregulated	^(35)^
		Ghrelin O-acyltransferase mRNA levels of the fundus in the stomach were higher in obese rats as compared with lean rats and had lowered gene expression in the OHF group	
		Food intake, satiety hormones and alterations in gut microbiota via which prebiotics acts were regulated in a dose-dependent manner	
4	Transcriptomics	Dietary JA supplementation significantly improved insulin resistance and hepatic triglyceride accumulation	^(36)^
		Transcriptomic profiling of the liver revealed that the expression of *malic enzyme 1, associated with fatty-acid synthesis; decorin, related to fibrosis; and cytochrome P450, family 1, subfamily a, polypeptide 2 and nicotinamide phosphoribosyl transferase* associated with inflammation was significantly improved by JA supplementation	
		10 % JA supplementation may be beneficial for the prevention of the onset of type 2 diabetes and non-alcoholic fatty liver disease	
5	Transcriptomics	Incorporation of KGM and inulin into the high-fat fiber-free diet beneficially reduced the malondialdehyde levels of the colon and liver and DNA damage in blood lymphocytes	^(37)^
		Antioxidative defense systems were enhanced by upregulating the gene expressions of colonic mucosa glutathione peroxidase and catalase and of hepatic superoxide dismutase and catalase	
		*In vivo* utilization of KGM and inulin activates both the local and systemic antioxidative defense systems in rats	
6	Transcriptomics	High-fat diet feeding significantly decreased the expression of regenerating islet-derived 3-gamma (*Reg3g*) and phospholipase A2 group-II (*PLA2g2*) in the jejunum	^(38)^
		Prebiotic treatment increased *Reg3g* expression and improved gut homeostasis as suggested by the increase in the expression of intectin, a key protein involved in gut epithelial cell turnover	
8	Proteomics	Low temperatures influence harvesting of chicory root, as 7 of the 21 most intense protein spots observed were associated with cold acclimation	^(43)^
9	Metabolomics	Inulin intake attenuated some of the HFD-induced metabolic changes in both myocardium (3-HB, lactate and guanosine) and testicle tissues (3-HB, inosine and betaine)	^(48)^
10	Metabolomics	Concentrations of esters, acids and some aldehydes in fecal samples from healthy volunteers were significantly increased with increasing doses of inulin	^(49)^
		Inulin dose-dependently inhibited the formation of S-compounds and the generation of other protein fermentation metabolites such as phenolic compounds	
11	Metagenomics of gut microbiota	Deep metagenomic sequencing analysis revealed that HFD and prebiotic treatment significantly affected the gut microbiota at different taxonomic levels	^(38)^
		Gut microbiota modulations induced by prebiotics counteracted HFD-induced inflammation and related metabolic disorders	
12	Genomics of gut microbes	Significant increase in *Faecalibacterium prausnitzii* after inulin intake	^(62)^
		The presence of *Bifidobacterium adolescentis* and *B. longum* in all volunteers, and *B. pseudocatenulatum*, *B. animalis*, *B. bifidum* and *B. dentium* was observed	
		*B. adolescentis* showed the strongest response to inulin consumption, significantly increasing from 0.89 to 3.9% of the total gut microbiota	
		Numbers of *B. bifidum* were remarkably increased from 0.22 to 0.63% in the five volunteers for whom this species was present	
13	Genomics of gut microbes	In pigs that fed on diets containing inulin, higher colonic bifidobacteria, lower total colonic SCFA concentrations due to reduced acetate but higher proportions of colonic butyrate were observed	^(63)^
		Inulin did not stimulate increase in lactobacilli and bifidobacteria numbers irrespective of the basal diet, although 20–50% of inulin was degraded in the jejunum	
14	Genomics of gut microbes	Consumption of prebiotic inulin/partially hydrolyzed guar gum mixture produced clinical results comparable to placebo in constipated females, but had additional protective effects on gut microbiota by decreasing the amount of pathological bacteria of the *Clostridium* genera	^(64)^
15	Genomics of gut microbes	In anaerobic fermenters containing different carbohydrate souces, including inulin, the growth of a greater proportion of *Bacteroides* and a lower proportion of Gram-positive anaerobes related to *Faecalibacterium prausnitzii*, *Ruminococcus flavefaciens*–*R. bromii*, *Eubacterium rectale*–*Clostridium coccoides* and *E. cylindroides* than the proportions in the starting fecal inoculum were supported	^(65)^
		Inulin from dahlia resulted in a significant increase in the number of bacteria related to *R. flavefaciens–R. bromii* and *E. cylindroides*	
16	Genomics of gut microbes	Inulin treatment had moderate effects on lactate, propionate and butyrate levels	^(66)^
		Denaturing gradient gel electrophoresis analysis revealed that inulin changed microbial metabolism by modulating the microbial community composition	
		Inulin has a lower potency than AXOS to shift part of the sugar fermentation toward the distal colon parts	
17	Genomics of gut microbes	A mixture of OF and OF-lcIN- and lcIN-containing diets resulted in larger numbers of cecal, colonic and fecal bacteria of the *C. coccoide–E. rectale* cluster.	^(67)^
		Higher amounts of lactobacilli were found in cecal and colonic contents of Mix OF-lcIN-fed rats and in feces of OF-fed rats	
		Mix OF-lcIN and OF resulted in significantly smaller numbers of cecal, colonic and fecal bacteria belonging to the *C. histolyticum* and *C. lituseburense* as compared with Con. Counts of *Bacteroides–Prevotella* and *Enterobacteriaceae* were similar between the groups	
		OF and/or lcIN-containing diets significantly increased the butyrate concentration and its relative molar proportion in the cecal and colonic contents	
		Only lcIN-containing diets resulted in a higher fecal concentration of butyrate than Con. Higher molar proportions of fecal butyrate were observed with all diets that had been supplemented with OF and/or lcIN	

Abbreviations: AXOS, arabinoxylan oligosaccharides; HFD, high-fat diet; JA, Jerusalem artichoke; KGM, konjac glucomannan; OHF, obese 20% fiber; OF, oligofructose; lcIN, long-chain inulin; SCFA, short-chain fatty acids.
